# Medication adherence in renal transplant recipients: A latent variable model of psychosocial and neurocognitive predictors

**DOI:** 10.1371/journal.pone.0204219

**Published:** 2018-09-28

**Authors:** Theone S. E. Paterson, Norm O’Rourke, R. Jean Shapiro, Wendy Loken Thornton

**Affiliations:** 1 Department of Psychology, Simon Fraser University, Burnaby, British Columbia, Canada; 2 Ben-Gurion University of the Negev, Beersheba, Israel; 3 University of British Columbia, Vancouver, British Columbia, Canada; Taipei Veterans General Hospital, TAIWAN

## Abstract

**Objective:**

Estimates indicate that 20–70% of renal transplant recipients are medication non-adherent, significantly increasing the risk of organ rejection. Medication adherence is negatively impacted by lower everyday problem solving ability, and associations between depressive symptoms, self-efficacy, and adherence are reported in renal transplant recipients. Nonetheless, to date, these associations have not been examined concurrently. Given the relationship between non-adherence and organ rejection, it is critical to gain a better understanding of the predictors of adherence in renal transplant recipients. To this end, we modeled relationships among cognitive abilities, depressive symptoms, self-efficacy, and adherence in this group.

**Methods:**

Participants (N = 211) underwent renal transplant at least one year prior to participation. Adherence was measured via self-report, medication possession ratio, and immunosuppressant blood-level. Traditionally-measured neurocognitive and everyday problem-solving abilities were assessed. Depressive symptoms were measured via self-report, as were general and medication adherence related self-efficacy. Structural equation modeling was used to assess the fit of the model to available data.

**Results:**

Everyday problem solving and self-efficacy had direct positive associations with adherence. Depressive symptoms were negatively associated with self-efficacy, but not adherence. Traditionally-measured neurocognitive abilities were positively associated with self-efficacy, and negatively associated with depressive symptoms.

**Conclusions:**

We present a comprehensive investigation of relationships between cognitive and psychosocial factors and adherence in medically stable renal transplant recipients. Findings confirm the importance of everyday problem solving and self-efficacy in predicting adherence and suggest that influences of depressive symptoms and neurocognitive abilities are indirect. Findings have important implications for future development of interventions to improve medication adherence in renal transplant recipients.

## Introduction

Failing to adhere to immunosuppressant medications by renal transplant recipients (RTR) is associated with elevated morbidity and mortality [1]. For instance, non-adherence is related to a sevenfold increase in rates of graft rejection [2]. Graft rejection can lead to loss of the transplanted organ and a return to end stage renal disease, necessitating return to dialysis, re-transplantation, or even causing death [1,3]. Despite potential dangers of failing to adhere to immunosuppressant medications, rates of medication non-adherence by RTR range between 20–70% [4].

Existing research has identified several demographic and psychosocial factors related to medication non-compliance in RTR [5,6]. For instance, social factors such as living alone and being unmarried have previously been associated with decreased adherence, as have perceived low level of social support, external locus of control, beliefs that medications are not required, and higher levels of anxiety and hostility [6]. However, another risk factor that remains relatively understudied to date involves the role of cognition. Indeed, relative to their healthy peers, adults with chronic kidney disease (CKD) demonstrate weaknesses in memory and executive functioning across the course of their illness [7,8] and following successful transplant [9,10]. These cognitive difficulties in CKD and RTR have been theorized to stem from cerebrovascular insufficiencies commonly reported in CKD and in dialysis patients that may not be reversed with transplantation [11].

Despite a paucity of research directly linking cognitive functioning and medication adherence in RTR, several studies provide indirect evidence. Self-reported unintentional non-adherence (forgetting) was identified as a probable source of non-adherence, with 62.4% of RTR reporting such difficulties [12]. In another study, RTR surveyed cited barriers to adherence that included difficulty reading medication instructions and remembering how many pills to take and when [13]. These findings suggest that cognitive difficulties such as forgetfulness, poor memory, and problems reading, understanding, or remembering instructions contribute to non-adherence in RTR. Nonetheless, despite subjective associations between cognition and adherence, none of the above studies with RTR objectively measured cognitive abilities (i.e., via structured neurocognitive assessments). Support for a relationship between cognitive functioning and adherence is, however, found in other chronic illness populations. For example, in persons receiving dialysis, non-adherent participants trend toward lower scores on a measure of global cognitive ability [14]. Further, in HIV+ persons, better learning and memory, executive functioning [15], and working memory have been associated with increased adherence [16].

Historically, cognitive functioning in chronic illness populations has focused on traditional neurocognitive abilities (i.e., learning, memory, and executive functions [7,8,10]). Notably, there is emerging interest in the role of "everyday" cognition, specifically, everyday problem solving (EPS) in health behaviors. While performance on EPS tasks may in part rely on traditional abilities such as executive function and episodic memory [17,18], EPS appears to represent a distinct aspect of cognition with additive utility in predicting real world outcomes, including self-rated functioning, life-skills functioning, and mortality [19–21]. Importantly, past research indicates that better EPS performance predicts better self-reported medication adherence in RTR [20]. Thus, not only must relationships between cognition and adherence be further examined, but the differential impacts of traditional neurocognitive abilities and EPS on medication adherence need to be established among RTR.

Another important factor to consider when examining adherence in RTR is depressive symptomatology. Rates of depression in RTR are reported to be nearly 3 times higher than the general US population (26% vs. 9.5%, respectively [22,23]). In fact, several studies have found that self-reported depressive symptoms are associated with medication non-adherence in RTR [12,20]. Previous research indicates that depression is not a unitary construct [24], yet research examining the various aspects of depressive symptoms with RTR is sparse [25].

Association between poor performance on neurocognitive tests and depressive symptoms are seen in other populations (e.g., traumatic brain injury [26], and older adults [27]). However, despite findings indicating that neurocognition and depressive symptoms vary together among dialysis patients and RTR (i.e., neurocognitive abilities are higher, and depressive symptoms lower, among RTR than dialysis patients [28]), few studies have examined associations between these two variables within RTR. In individuals with CKD however, negative relationships are seen between performance on executive functioning tasks and depressive symptoms [29]. Previously published research from our laboratory also suggests that decreased EPS performance, along with increased depressive symptoms, predicts lower adherence in RTR [20].

A related factor that is potentially critically important to medication non-adherence is self-efficacy. Self-efficacy can be considered the antithesis of helplessness, a core facet of depressive symptomatology [30]. Perceived self-efficacy involves one’s ability to change and adapt by influencing how they envision their abilities and the limits of their capability [31]. Self-efficacy can be examined generally by assessing willingness or ability to initiate behavior, put effort into completing behaviors, and one’s persistence when faced with adversity [32]. Researchers have also developed behavior-specific measures of self-efficacy, including those focusing on adherence-related behaviors [33].

Existing research demonstrates links between self-efficacy and non-adherence in end stage renal disease and RTR. For instance, both general [34], and adherence-related [35] self-efficacy have been associated with treatment adherence in hemodialysis patients. Similarly, higher adherence-related self-efficacy perceptions have been associated with better compliance with a low fluid intake protocol for dialysis patients [36], and better medication adherence among adult [37] and pediatric [38] RTR. Second only to side-effects complaints, Rudman and colleagues [3] report that adherence-related self-efficacy significantly predicts treatment compliance in RTR. Despite indications in end stage renal disease that both general and specific self-efficacy appraisals are associated with treatment adherence, the relative contributions of general versus adherence-related self-efficacy in predicting medication adherence in RTR have not been examined to date.

Importantly, the aforementioned link between non-adherence, cognition and depressive symptoms may be at least partially explained by variations in self-efficacy. Previous findings suggest that self-efficacy may mediate the association between neurocognitive abilities and adherence in RTR [39]. Higher self-efficacy has also been linked to lower depressive and somatic symptoms, pessimism, and interpersonal vulnerability in RTR [40,41]. Research with other populations including hemodialysis patients [35] indicates that self-efficacy may mediate the relationship between depressive symptoms and adherence [42]. Thus, self-efficacy may account (in whole or in part) for associations between these cognitive and affective variables and non-adherence in RTR.

To address these issues and advance our understanding of the interplay between these psychosocial and neurocognitive variables in RTR, the current study examined the interrelations of medication adherence (in the present study, specifically implementation adherence—defined here as the extent to which an individual’s actual dosing reflects their prescribed medication regimen [43]), depressive symptoms, self-efficacy, EPS, and neurocognitive abilities to clarify relationships between these variables previously only examined via univariate methods. This multivariate approach allowed us to simultaneously model our four latent independent variables, interrelatedness among these latent variables, and the relative importance of each as a predictor of medication adherence in RTR. Our multivariate model includes neurocognitive abilities (i.e., intelligence, executive functions, and memory), EPS, depressive symptoms (depressed affect, absence of well-being, somatic symptoms, and interpersonal rejection), and self-efficacy (both general and adherence-related appraisals), to examine direct and indirect associations between these variables and medication adherence. To our knowledge, this represents the first comprehensive multivariate investigation of cognitive abilities, depressive symptoms, and self-efficacy in relation to adherence in a sample of RTR.

**Fig 1:**
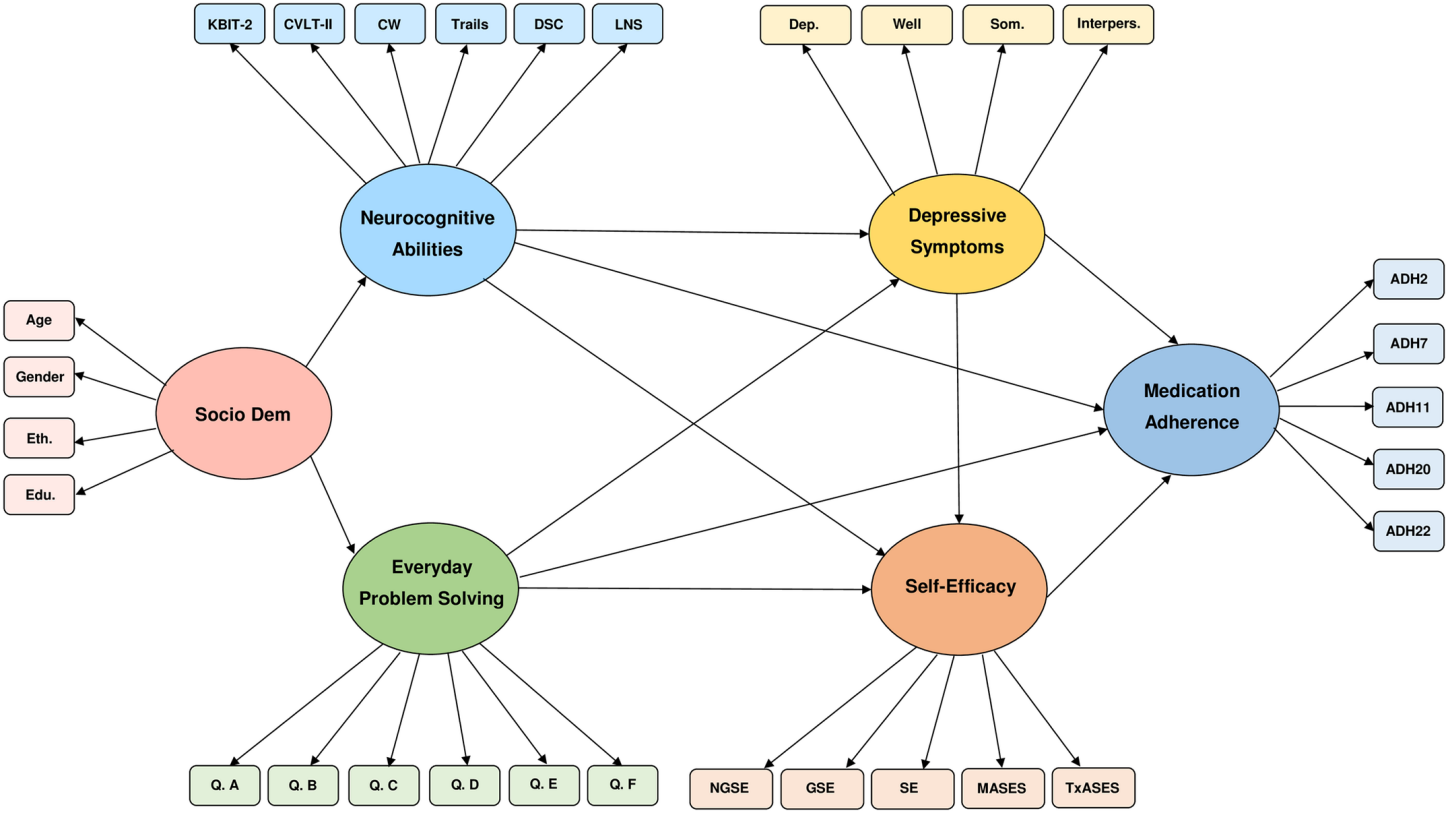
Initial estimated model of adherence by renal transplant recipients. Edu. = years of education, Eth. = ethnicity, Socio Dem = Socio-demographic latent variable, Dep. = CES-D Depressed affect, Well = CES-D Absence of Wellbeing, Som. = CES-D Somatic Symptoms, Interpers. = CES-D Interpersonal Rejection, SE = Self-Efficacy scale, GSE = General Self-Efficacy scale, NGSE = New General Self-Efficacy Scale, MASES = Medication Adherence Self-Efficacy Scale—Revised, TxASES = Transplant—Adherence Self-Efficacy Scale, ADH2 = TxEQ Adherence question 2, ADH7 = TxEQ Adherence question 7, ADH11 = TxEQ Adherence question 11, ADH20 = TxEQ Adherence question 20, ADH22 = TxEQ Adherence question 22, LNS = WAIS Letter-number sequencing, DSC = WAIS-Digit Symbol Coding, Trails = Trails Number-Letter Switching, CW = Color-Word Inhibition, CVLT-II = CVLT—Long Delay Free Recall, KBIT-2 = KBIT-2 IQ composite, Q. A through Q. F = EPS Test questions A through F.

We hypothesized that better performance on neurocognitive and EPS measures would predict better adherence. Also consistent with past work in RTR and other clinical populations [20,42], we hypothesized that depressive symptoms would directly, negatively, predict this type of adherence in RTR. In line with documented associations between self-efficacy and adherence [37], we hypothesized that self-efficacy would directly predict adherence. Finally, given evidence of the mediating role of self-efficacy in the association of adherence with depression [42] and aspects of cognition [39], we hypothesized that self-efficacy would mediate associations between neurocognitive abilities, EPS, depressive symptoms, and adherence (Fig 1). Given that both general and adherence-specific self-efficacy were measured, we also hypothesized that general self-efficacy would be of greater relative importance as general self-appraisals are thought to better reflect trait features than adherence specific appraisals [44].

## Materials and methods

### Participants

**Fig 2:**
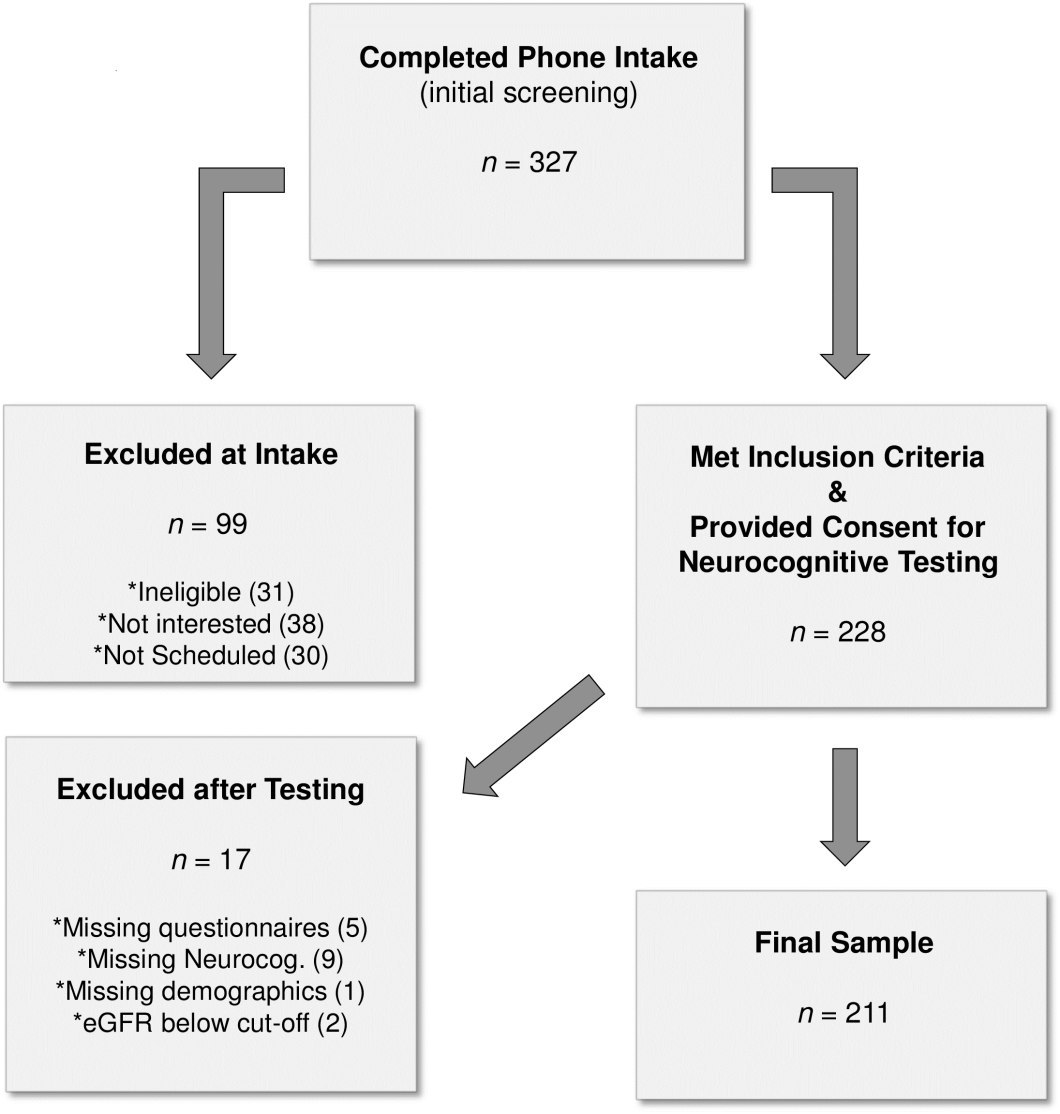
Participant recruitment flow chart. Note: Regarding inclusion/exclusion criteria at intake, participants who indicated less than 3 of 4 preferences as “English” for speaking, reading, writing, and thinking, on an acculturation questionnaire were considered ineligible due to language requirements for neurocognitive testing; in addition, those reporting any of the abovementioned physical or mental health exclusions were ineligible; Not interested = participants who declined study participation due to lack of interest; Not scheduled = participants who expressed interest in the study, but who had scheduling conflicts, and did not participate.

Between 2011–2014, 228 participants were recruited from the Solid Organ Transplant Clinic at Vancouver General Hospital, Vancouver (BC), Canada. Eligible, participants met the following criteria: 1) successful kidney graft at least one year prior to recruitment; 2) adequate vision (i.e., equal to or better than 20/70, corrected or not [45]) and hearing (corrected or not), and free of other sensory impairments that would significantly interfere with testing; 3) sufficient English fluency; 4) education of at least a 6th grade level to ensure adequate comprehension of written materials; 5) stable renal functioning determined by estimated glomerular filtration rate (eGFR, an estimation of filtration capacity of the functioning nephrons and measure of kidney reserve) above 14ml/minute per 1.73 m2. Exclusion criteria included the diagnosis of a psychotic disorder, acute physical illness, a diagnosed neurological disorder (e.g., Parkinson’s disease), or any other major organ failure (e.g., end stage liver disease). A flow chart describing participant recruitment and participation is presented in Fig 2.

Prospective participants were mailed study invitation letters and follow-up phone calls to interested participants were made by trained research assistants. All participants signed letters of informed consent and received $30. Testing conducted individually, by trained examiners, required approximately 1.5–2 hours. This study was approved by the Simon Fraser University Research Ethics Board (dore@sfu.ca; application number 2010s0574), and by the University of British Columbia (https://ethics.research.ubc.ca; application number H04-80470) and the Vancouver Coastal Health Authority (www.vchri.ca; application number V04-0252).

### Measured variables

#### Demographics and clinical data

All measures were administered by trained research assistants. Information on various illness variables was collected via self-report and existing medical records. Participants reported their age, gender, level of education, ethnicity, and living situation. Health variables included time since transplant, number of transplants received, donor type (living or cadaveric), CKD stage, time on dialysis prior to transplant, CKD diagnosis, history/current diagnosis of diabetes, and eGFR and hemoglobin levels (g/L; low levels are indicative of anemia).

#### Depressive symptoms

The *Center for Epidemiological Studies Depression Scale* (CES-D [46]) is a commonly used 20-item scale measuring the frequency of various depressive symptoms. Factor analyses with various populations (including pre and post renal transplant) have identified four separate factors labeled Depressed Affect, Absence of Well-Being, Somatic Symptoms, and Interpersonal Rejection that contribute significantly to measurement of a higher-order depression construct [24,25,47,48]. These individual factors were retained in the current study to allow examination of the role of each in predicting adherence.

#### Self-efficacy

We utilized well-established measures of both General self-efficacy and Medication Adherence specific self-efficacy. General self-efficacy was assessed with three different self-report measures, so as to provide multiple discrete observed variables for inclusion within the hypothesized self-efficacy latent construct, given that latent variables in SEM should ideally include at least 3 observed variables [49]. The 30-item *Self-Efficacy Scale* was developed by Sherer and colleagues [32] to evaluate general and social self-efficacy. *The General Self-Efficacy Scale* (GSE [50]) was created to assess a general sense of perceived self-efficacy to predict coping with daily hassles and adaptation to various types of stressful life events. *The New General Self-Efficacy Scale* (NGSE [51]) was created to provide a more concise and valid update to the general self-efficacy subscale of the abovementioned *Self-Efficacy Scale* published by Sherer and colleagues [32].

Medication adherence-specific self-efficacy was measured with two self-report questionnaires. The *Adherence Self-Efficacy Scale* (ASES [52]) was slightly modified for RTR research, with one HIV-specific item removed. Respondents rate their efficacy to carry out important treatment-related behaviors including medication adherence in the face of possible barriers. The *Medication Adherence Self-Efficacy Scale—Revised* (MASES-R [33]) asks individuals to assess their ability to carry out important medication adherence-related actions when faced with various possible stresses.

#### Neurocognitive measures

Well-established tasks assessing multiple cognitive domains were utilized to provide measurement of aspects of cognition commonly examined in RTR [7,8,10]. The *Kaufman Brief Intelligence Test* (KBIT-2 [53]) assesses verbal and non-verbal intellectual functioning. The Verbal scale contains both a Verbal Knowledge task (assessing general information and word knowledge), and the Riddles task (assessing verbal concept formation). The Nonverbal scale consists of the Matrices task (assessing nonverbal reasoning). The *California Verbal Learning Test* (CVLT-II [54]) was used to assess verbal memory. The Long-Delay Free Recall [LDFR] score was examined, providing an estimate of participants’ ability to retain verbal information. The *Delis–Kaplan Executive Function System* (D-KEFS [55]) assesses aspects of executive functioning. Trial 4 (Letter-Number Sequencing) of the *Trail Making Test* of this battery is used as a measure of set-shifting [56], and Trial 3 (Inhibition) of the *Color-Word Interference* task is used as a measure of cognitive inhibition [57]. The *Digit Symbol-coding subtest* of the *Wechsler Adult Intelligence Scale III* (WAIS-III [58]) is a measure of processing speed, with the total number of correctly matched items within 120 seconds as the outcome measure. Working memory was assessed with the *Letter-Number Sequencing subtest* (WAIS-III [58]), with the number of successively longer reordered number-letter spans produced as the outcome variable.

#### Everyday problem-solving

The *Everyday Problem Solving* (EPS) task consists of six paper and pencil vignettes used in previous research [18–20,59,60]. Participants are instructed to read each problem carefully, record as many solutions as possible, and include all solutions, even those that they themselves would not consider using. Problems are structured to allow for generation of multiple responses (e.g., “An elderly man has just retired. He doesn’t have any hobbies because he has never had time for them before. Now he is really bored. What should he do?”), and the number of safe and effective solutions generated was used as the outcome measure. Inter-rater agreement seen in previous research among CKD patients, using these scoring criteria, has been very high (e.g., *r*_*ic*_ = .96 [18]). Inter-rater reliability was similarly high in the current study (r_ic_ = .94).

#### Medication adherence

Prior to cognitive assessment, participants’ three most recent cyclosporine/tacrolimus serum levels were noted from medical records. Serum immunosuppressant levels were classified as “at target level” if two or three of three levels were within the target range for that medication. They were classified as ‘not at target level’ if less than two levels were within target range (450 ng/mL or higher at 2 hours (C2) post dose for cyclosporine; 4–8 ng/mL measured on the trough (or c0) level for tacrolimus). Transplant recipients followed at the Solid Organ Transplant clinic undergo routine medication serum level monitoring on approximately a monthly basis, so information collected generally reflects the three-month period prior to neurocognitive assessment.

Pharmacy refill data for participants’ primary immunosuppressant medication was used to calculate a medication possession ratio (MPR), indicating the percentage of prescribed medication an individual obtained within a specified period, compared to that which they should have obtained during that period.

Participants completed the *Adherence* subscale of the *Transplant Effects Questionnaire* (TxEQ [61]) to assess how well they were adhering to their medication regimen (e.g. “Sometimes I forget to take my antirejection medicines”). Items are endorsed on a 5-point Likert type scale (“Strongly Agree” to “Strongly Disagree”) with total score range of 5–25 for this subscale, and higher scores indicating better adherence [61]. Work by Frazier and colleagues indicates that measurement of non-adherence is improved by use of a continuous measure, which unlike many self-report scales utilizing ‘yes’ or ‘no’ responses, the TxEQ allows [62]. Similarly, previous research indicates that self-report measures that ask for qualitative reports of adherence better approximate adherence level as measured by electronic monitoring than those querying specification of missed doses [63].

In a systematic review, the TxEQ Adherence scale has been shown to have good reliability and established content validity [64]. Test-retest reliability of responses to this scale after a 1-month interval is high (*r* = .77 [61]). Internal consistency of this scale is also satisfactory (α = .78), while convergent and divergent validity analyses demonstrate correlations between conscientiousness, neuroticism, emotional coping, and the *Adherence* scale [65]. Responses to the five TxEQ Adherence items have also been shown to significantly correlate with quality of life related to health in RTR [66].

### Data analyses

#### Statistical methodology

Structural equation modeling (SEM) was used to test the mathematical viability of an omnibus multivariate model of predictors of treatment adherence among individuals greater than one year post renal transplant. Neurocognitive Abilities, Everyday Problem-Solving, Depression, Self-Efficacy, and Medication Adherence served as latent variables represented by scores on the specific (observed) measures described above. As SEM is a confirmatory form of analysis, use of this technique allowed us to test the theorized causal model seen in Fig 1. Various goodness of fit indices are used to assess model fit to data in structural equation modeling. As recommended by O’Rourke and Hatcher [49], we report incremental, absolute, and parsimonious goodness of fit indices.

The Comparative Fit Index (CFI) provides a measure of incremental or relative fit. The CFI is thought to be the best index in covariance structure analyses, with little sampling variability for a relative fit index [67]. Values greater than .94 indicate good fit between the proposed model and actual data [68].

The Root Mean Square Error of Approximation (RMSEA) is a parsimony index and Standardized Root Mean Square Residual (SRMR) is an absolute fit index. For both, values less than .09 indicate adequate fit [69] and values less than .055 indicate good fit [49]. The 90% confidence interval is also often reported for the RMSEA, with a related test referred to as *p* of Close Fit (pCLOSE). This latter measure is a test of the null hypothesis that the RMSEA is .05. As such, a non-significant result on this test indicates a “close” fit of the proposed model [69]. The SRMR measures the difference between observed and predicted correlation.

#### Model development

The goal of this study was to provide an examination of the relationships between latent constructs as opposed to an in-depth examination of the separate aspects of neurocognitive abilities, depression and self-efficacy. Therefore, all of our neurocognitive measures were included on a single latent variable, a decision supported by previous research examining neurocognitive functioning in RTR that has shown significant covariance among neurocognitive tasks in this population [20], Similarly, all aspects of depression, and types of self-efficacy measured, were each included on a single latent variable, respectively.

Variables previously associated with adherence in solid organ transplant research were considered for inclusion on a prospective socio-demographic latent variable to account for additional impact these variables may have on adherence. Correlations were examined between our adherence measures (TxEQ Adherence, immunosuppressant target, and MPR) and possible covariates including age, living situation (alone vs. with others), level of education, gender [6], ethnicity (Caucasian vs. non-Caucasian [70]), donor type (relative vs. unrelated [71]), time since most recent transplant, and level of employment (more or less than 20 hours per week [72]). Significant correlations were seen only for gender and ethnicity. Although neither age nor level of education correlated with any of our three adherence measures in these preliminary analyses, these two covariates were also initially retained in the originally proposed model due to their impact on neurocognitive and EPS measures in previous research [9,18].

#### Confirmatory factor analyses

As an initial step in testing our model, we used confirmatory factor analyses to test the viability of measurement models for each of our latent variables without causal paths between them. For all latent constructs described above, measured variables loaded onto their respective latent variables, with the exception of the adherence latent construct. For the conceptualized adherence construct (i.e., five items that make up the TxEQ Adherence scale, immunosuppressant target, and MPR) the latter two variables did not load onto the same factor as the five TxEQ items. As a result, target level and MPR were removed and placed on a second adherence variable; however, these two variables again did not load onto the same factor and were thus removed from the subsequent SEM analyses. The CFI and RMSEA of the final adherence latent variable were optimal (CFI = 1.00; RMSEA = .005; Table 1 shows lack of significant correlations between the adherence variables). Fit indices for the remaining latent variables were all acceptable (EPS: CFI = .99; RMSEA = .052; depression: CFI = 1.00; RMSEA < .001; self-efficacy: CFI = 1.00; RMSEA < .001; traditional neurocognition: CFI = .98; RMSEA = .071).

**Table 1:** Correlation Matrix for adherence variables.

	**Target**	**MPR**	**TxEQ**
Target	--		
MPR	-.019	--	
TxEQ	.042	.125	--

*p* * < 0.050, (There are no significant correlations).

Given the results of the CFA, immunosuppressant target level and MPR data were not included in the tested model, which relied instead on a previously validated self-report adherence measure shown to have good reliability in transplant samples [73]. The inability of the three adherence measures to load on the same construct highlights discrepancies in what these methods measure. Previous research suggests mixed results with respect to agreement between measurement methods [20,74,75]. The fact that each of these measures captures a different facet of adherence (i.e., blood level of medication, medication used over a period, self-reported estimates of adherence behaviors) may explain non-significant correlations between measures in this and other samples.

With this in mind, previous research indicates that self-report measures better approximate adherence as measured via electronic pill-top monitoring devices than do other forms of adherence assessment [2], and provide the most conservative estimate of adherence in RTR according to meta-analytic review (i.e., highest rates of non-adherence) [70]. Unlike the other measures of adherence considered in the present study, measures that rely on self-report are not affected by medication half-life and may, unlike refill data, provide an indication of whether medications that were picked up, were ingested.

Pitfalls of immunosuppressant blood level and MPR measures have been shown to include individuals’ increasing adherence behaviors just prior to clinic visits where blood is taken, or stockpiling medications retrieved from the pharmacy [76]. MPR data in the present study are at ceiling (M = .99), supporting assertions that this method can overestimate adherence [76]. Serum level measurements may also underestimate adherence due to half-life of the medication [77], and are further impacted by differences in pharmacodynamics and pharmacokinetics of medications both within and between individuals, type of analytical methods used to determine serum concentration, and potential for interactions between multiple medications, with dietary factors, and with comorbid diseases [77,78].

## Results

### Descriptive statistics

The study sample was 59.7% male, with significantly more men than women (*t*[210] = 41.45, *p* < .001). Of note, 20% of the sample scored above the CES-D threshold value suggestive of clinically significant depressive symptoms. Approximately 90% of participants were in stages 1–3 of CKD based on eGFR levels, indicating they may have at most some mild symptoms including tiredness, poor appetite, and/or itching associated with kidney dysfunction [79]. Fewer than 10% were in stage 4, in which patients commonly experience more pronounced physical symptomatology (see Table 2). No differences in adherence were seen between those with first vs. second transplants, or those who received living vs. deceased donor transplants. Time since transplant ranged from 1.17 to 30.5 years, with 15.6% of the sample having received their transplant within the previous 3 years.

**Table 2:** Sample demographic and measurement characteristics.

**Participant Characteristics**	**Study Sample (*N* = 211)**	
Age	53.93 ± 11.91	
Male	126 (59.7%)	
Ethnicity		
*Caucasian*	*137 (64*.*9%)*	
*Asian*	*41 (19*.*4%)*	
*Other*	*33 (15*.*7%)*	
Education	13.96 ± 2.35	
Living Situation		
*Alone*	*30 (14*.*2%)*	
*With Spouse*	*147 (69*.*7%)*	
*With Other*	*34 (16*.*1%)*	
Distance from Clinic		
*<1 mile*	*8 (3*.*8%)*	
*1–10 miles*	*71 (33*.*6%)*	
*10–30 miles*	*94 (44*.*5%)*	
*30–100 miles*	*37 (17*.*5%)*	
Number of Renal Transplants (> 1)	21 (10.0%)	
Transplant type (deceased donor)	102 (48.6%)	
Time Since Transplant (years)	8.98 ± 6.74	
Time on Dialysis Pre-transplant (years)	2.34 ± 2.42	
Hemoglobin Level (gm/L)	131.38 ± 17.39	
eGFR Level (ml/min)	56.42 ± 19.74	
CKD Stage		
*1 (eGFR ≥ 90)*	*13 (6*.*2%)*	
*2 (eGFR = 60–89)*	*76 (36*.*4%)*	
*3 (eGFR = 30–59)*	*100 (47*.*8%)*	
*4 (eGFR = 15–29)*	*20 (9*.*6%)*	
**Study Measures**	***M* (*SD*)**	***N (%)***
CES-D	9.76 (8.39)	
CES-D Score >15		44 (20.9%)
GSE	32.79 (4.82)	
SE	85.35 (12.44)	
NGSE	31.89 (5.55)	
Tx-ASES	101.15 (11.70)	
MASES-R	49.26 (4.66)	
KBIT-2	101.49 (13.13)	
DKEFS CW	10.67 (2.80)	
DKEFS Trails	10.24 (3.15)	
CVLT-II	.41 (1.06)	
WAIS LNS	10.86 (3.01)	
WAIS DSC	8.35 (3.05)	
EPS	24.03 (7.72)	
TxEQ	21.26 (3.79)	
MPR	.99 (.12)	
Target (on target)		161 (85.2%)

*Note*: Mean (*M*) and standard deviation (*SD*) for continuous variables; frequency (*N*) and percentage (*%*) for dichotomous variables. Higher scores = better performance (except CES-D—higher scores indicate increased symptoms).

CES-D = Center for Epidemiological Studies Depression Scale (range: 0–60; ≥ 16 = clinically significant symptomatology). GSE = General Self-Efficacy Scale (range: 10–40); SE = Self-Efficacy Scale (range: 30–150); NGSE = New General Self-Efficacy Scale (range 8–40); Tx-ASES = Transplant—Adherence Self-Efficacy Scale (range: 11–110); MASES-R = Medication Adherence Self-Efficacy Scale—Revised (range: 13–52).

For traditional neurocognitive measures, standard (*M* = 100, *SD* = 15), scaled (*M* = 10, *SD* = 3), or Z-scores (*M* = 0, *SD* = 1) are reported; study *M* (*SD)* indicated. KBIT-2 = KBIT-2 IQ Composite score; D-KEFS CW = D-KEFS Color-Word Interference Trial 3; D-KEFS Trails = D-KEFS Trail Making Test Trial 4; CVLT-II = CVLT-II Long Delay Free Recall; LNS = WAIS-III Letter Number Sequencing; DSC = WAIS-III Digit Symbol-Coding.

EPS = Everyday Problem Solving test (theoretical range: 0-infinite); TxEQ = Transplant Effects Questionnaire—Adherence (range: 5–25); MPR = medication possession ratio: Target = cyclosporine/tacrolimus serum on/off target (dichotomous).

Fifteen participants were removed from the SEM analyses due to missing data (14 with substantial non-random missing data; 1 missing demographic data that could not be imputed). For included participants, missing data were minimal for most variables examined (generally 1–2 missing values per variable). Multiple imputation was used to provide complete data for Maximum Likelihood methods in AMOS [80]. Results were confirmed using Full Information Maximum Likelihood methods to allow for missing data.

### Estimation of initial model

The baseline model included some non-significant paths. After correcting for correlations between 30 pairs of error terms, model fit was improved, χ^*2*^[*df* = 365] = 601.326, *p* < .001. However, some of the fit indices suggested the model was not a good fit to the data. The RMSEA indicated adequate fit (RMSEA = .056, CI: .047-.063; pCLOSE = .13), but values for the CFI (.88) and the SRMR (.097) fell below ideal limits. Also of note, originally hypothesized pathways between neurocognitive abilities and self-efficacy, neurocognitive abilities and adherence, EPS and self-efficacy, and depressive symptoms and adherence were not statistically significant. Additionally, none of the four socio-demographic variables loaded onto that latent variable, and the pathways from the socio-demographic variable to traditional neurocognition and to EPS were not significant.

### Revision and estimation of final model

Revisions were made to improve model fit to the data. Age and gender were removed from the socio-demographic latent variable, leaving education and ethnicity, and with these changes, pathways from the modified socio-demographic variable (accounting for ethnicity and education) to neurocognition and EPS were significant. To ensure the best model fit, a specification search was conducted in AMOS with nonsignificant paths between latent constructs included in the original model considered as optional paths. Parameters for alternative models were examined to determine which paths should and should not be removed. The path from neurocognitive abilities to self-efficacy was retained, however when this model was examined, this path remained nonsignificant, and the path between EPS and depression was also nonsignificant. A subsequent specification search was conducted with these two paths considered optional, and the most parsimonious model was that in which the path between EPS and depression was removed. This model was again examined and the path between neurocognitive ability and self-efficacy was significant, and thus retained within our final model. It is possible that given the relationship between depression and self-efficacy, the path from EPS to depression had a suppressing effect on the relationship between neurocognitive abilities and self-efficacy in our original model.

**Fig 3:**
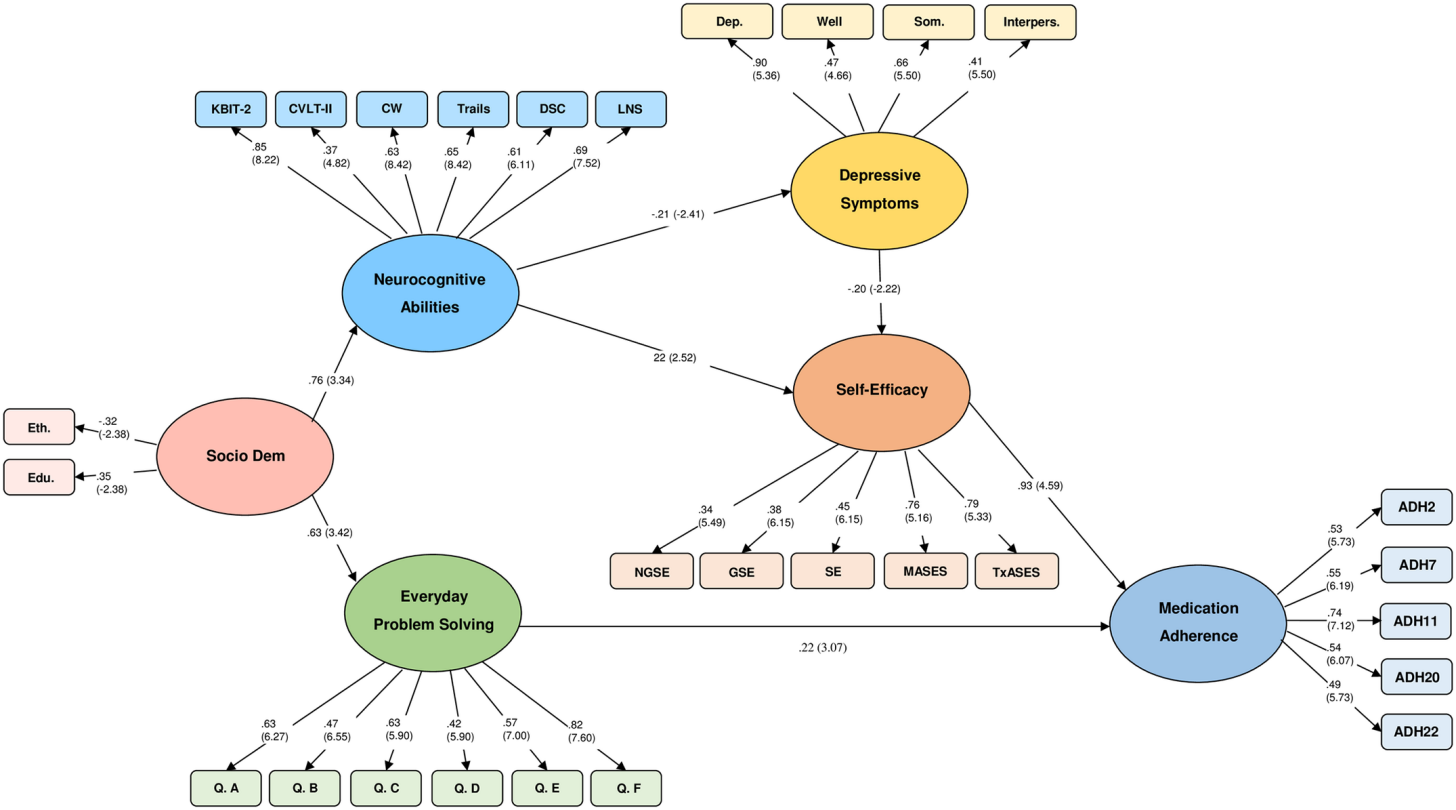
Final revised model of medication adherence among renal transplant recipients. Parameters are noted as standardized regression weights. Statistically significant CR values (in parentheses) are greater than |1.96|.

In our final model, both EPS and self-efficacy directly positively predicted medication adherence, while neurocognitive abilities and depressive symptoms did not. Neurocognitive abilities did both positively predict self-efficacy and inversely predict depressive symptoms, while depressive symptoms inversely predicted self-efficacy, indicating both direct and indirect relationships between neurocognitive abilities and self-efficacy (Fig 3). All paths that remained between latent constructs were statistically significant, and all parameter estimates were statistically significant. Based on the formula first reported by Maccallum and colleagues [69], statistical power for the resulting model was estimated at .99. Given the multiple hypotheses tested in the present model, we used the Benjamini-Hochberg [81] method of controlling false discovery rate (FDR) with adjusted *p*-values calculated via a spreadsheet program developed by McDonald [82]. All evaluated hypotheses remained significant.

Goodness of fit indices indicated acceptable fit between data and the revised model, χ*2*(*df* = 313) = 376.24, *p* = .009. The SRMR (.072) is satisfactory, whereas values for the CFI (.97), and the RMSEA (.031) are both ideal. Moreover, the full range of the 90% confidence interval for the RMSEA was within ideal parameters (.017-.042), indicating that the hypothesis of a non-close fit can be rejected (PCLOSE = 0.99 [69]).

## Discussion

To these authors’ knowledge, these findings represent the first approach to modeling concomitant relationships between medication adherence (i.e., implementation adherence) and relevant cognitive, affective, and psychosocial factors with RTR in medically stable transplant recipients. This model highlights the importance of EPS ability and self-efficacy appraisals in predicting self-reported medication adherence. The model also provides important insights in relation to indirect relationships between neurocognitive abilities and depressive symptoms, respectively, and self-reported adherence, suggesting that the respective influences of these variables on adherence are not as direct as has generally been reported by the extant literature to date.

Our results provide important insights into the interrelations between variables related to longer term adherence that have previously only been examined independently of one another. In the context of the current model, both self-efficacy and EPS directly positively predict self-reported adherence. Neither depressive symptoms nor neurocognitive abilities are directly associated with adherence. While EPS is directly related to adherence (as in previous research [20]), self-efficacy mediates the relationship between neurocognitive abilities and adherence. This mediating effect of self-efficacy indicates the important role of self-efficacy in predicting adherence, whereas neurocognitive abilities appear to have a more indirect role in medication adherence (via self-efficacy and depressive symptoms). As research examining these variables is limited to date, these findings represent an important step forward in our understanding of the relationships between cognition, self-efficacy, and adherence in RTR.

Interestingly, measures of adherence-related self-efficacy were seen to have higher loadings on the self-efficacy latent construct than general self-efficacy measures, suggesting that adherence-related measures make a greater contribution to the self-efficacy construct among RTR. Given the importance of self-efficacy in the prediction of adherence, future research should focus on examining the relative impact of both general and adherence-related self-efficacy on medication adherence in RTR.

The relationships observed between neurocognitive abilities and EPS with adherence highlight important differences in the predictive utility of these measures for adherence in individuals who have been transplanted for over a year. EPS predicts self-reported adherence directly and independently from the effects of all other considered variables in the current sample. The EPS measure used in this study requires participants to generate multiple safe and effective solutions to problems (see [83] for a review). As such, better performance on this task is thought to reflect the ability to generate multiple efficacious solutions to problems that may arise (e.g., how to avoid missing a dose of medication when out of the house, when at work, when feeling ill, and under other circumstances). Future research examining EPS in relation to adherence in RTR and other groups is warranted. The roles of self-efficacy and EPS also pinpoint potential targets for future interventions to increase medication adherence across the lifespan.

The absence of a direct effect of depressive symptoms on adherence ran counter to the hypotheses. In the revised model, depressive symptoms were negatively related to self-efficacy, which in turn, was directly, positively, related to self-reported adherence. The path through self-efficacy between depressive symptoms and adherence highlights the importance of self-efficacy ratings in the prediction of adherence. Past findings of associations between depressive symptoms and adherence have failed to account for this influence of self-efficacy [20]. In our revised model, direct relationships were also observed between neurocognitive abilities and both depressive symptoms and self-efficacy, indicating that better cognitive abilities are associated with both higher self-efficacy and lower depressive symptoms. Consistent with past research [23], 21% of the study sample scored above a clinical cut-off for depression, thus, relationships between these variables deserve increased attention going forward.

Understanding the interplay between cognition, depressive symptoms, and self-efficacy appraisals in impacting self-reported medication adherence one year or more post-transplant will better enable practitioners to flag individuals at risk for non-adherence over the longer term. This can also enable follow-up care by psychologists or social workers who may be able to provide targeted assessment of cognition, mood and/or adherence-related self-efficacy, as well as applicable interventions tailored to each RTR’s specific challenges. Further, these findings highlight the need for development and implementation of interventions that focus on increasing general and adherence-related self-efficacy, improving/compensating for EPS difficulties, and decreasing depressive symptoms. Research is needed to develop novel interventions to improve adherence, and effectually, real-world function and quality of life post-transplant. Future research should also focus on development and piloting of predictive adherence measures utilizing EPS and self-efficacy appraisals, with focus on easily administered measures that can be utilized by health care practitioners.

### Limitations and conclusions

Limitations of the present study warrant mention. First, the study sample was derived from one transplant center in western Canada. It will be important to replicate findings in other groups of RTR from different regions in Canada, the United States, and abroad, to examine whether findings generalize to other samples.

The cross-sectional nature of this study also precludes definitive conclusions regarding the causality of observed relationships. Many of the variables examined in the present study may fluctuate for an individual across time (e.g., level of depressive symptomatology, level of self-efficacy both in general and in relation to adherence), as such, longitudinal studies are needed to examine how these constructs vary in relation to one another over time. Time since transplant in our sample was also greater than one year, with a focus on variables of possible impact on adherence across the lifespan as opposed to factors which may preferentially impact acute adjustment to adherence regimen post-transplant. The current model is thus likely not accurate to the initial adjustment phases of adherence post-transplant, and future research testing this model with samples at various time-points post-transplant would be beneficial. Additionally, availability of participants precluded the inclusion of an additional hold-out sample with which to externally assess our final multivariate model. As such, these analyses should be considered exploratory, and for this reason as well, should be confirmed with another sample of RTR.

Finally, as noted above, the model did not include immunosuppressant target level or MPR data, relying on self-reported adherence. As discussed, the failure of these distinct measures to load on one construct highlights dissociations and mixed agreement between these forms of measurement previously reported in the literature [20,71,72], likely related to the fact that each captures a different facet of adherence (i.e., blood level, medication collected, self-reported behavior). Although previous research has indicated that self-report measures better approximate adherence as measured via electronic pill-top monitoring devices than do other forms of adherence assessment [2], and provide the most conservative estimate of adherence in RTR [70], thus justifying use of self-report measures in adherence research, future research should certainly aim to reconcile these different measurement methods. Future research that examines models of relationships between immunosuppressant level data and MPR measures of adherence, respectively, and cognitive and psychosocial constructs, are an important next step given the present results. It will be important to determine whether similar relationships are seen between cognitive and psychosocial variables and these different types of adherence measures in RTR. Additionally, it will be important to replicate these findings using other self-report measures of adherence to confirm the relationships seen in the present study.

## Supporting information

S1 Appendix List of Acronyms.docx. (DOCX)
