# Supplementary material for: Medication adherence in renal transplant recipients: A latent variable model of psychosocial and neurocognitive predictors
Source: PLoS One. 2018 Sep 28;13(9):e0204219. doi: 10.1371/journal.pone.0204219 (PMC6161882; doi:10.1371/journal.pone.0204219)
Supplement: S1 Appendix — (DOCX) [file pone.0204219.s001.docx]

List of Acronyms

| ASES | Adherence Self-Efficacy Scale |
| --- | --- |
| CES-D | Centre for Epidemiological Studies Depression Scale |
| CFI | Comparative Fit Index |
| CKD | Chronic Kidney Disease |
| CVLT-II | California Verbal Learning Test, 2^nd^ Edition |
| D-KEFS | Delis-Kaplan Executive Functioning System |
| eGFR | Estimated Glomerular Filtration Rate |
| EPS | Everyday Problem-Solving |
| GSE | General Self-Efficacy Scale |
| KBIT-2 | Kaufman Brief Intelligence Test, 2^nd^ Edition |
| MASES-R | Medication Adherence Self-Efficacy Scale - Revised |
| MPR | Medication Possession Ratio |
| NGSE | New General Self-Efficacy Scale |
| RMSEA | Root Mean Square Error of Approximation |
| RTR | Renal Transplant Recipients |
| SEM | Structural Equation Model |
| SRMR | Standardized Root Mean Squared Residual |
| TxASES | Transplant Adherence Self-Efficacy Scale |
| TxEQ | Transplant Effects Questionnaire – Adherence Subscale |
| WAIS-III | Wechsler Adult Intelligence Scale, 3^rd^ Edition |
